# Perioperative antiplatelet in elderly patients aged over 70 years treated with proximal femur fracture: continue or discontinue?

**DOI:** 10.1186/s12891-019-2504-5

**Published:** 2019-03-25

**Authors:** Chul-Young Jang, Dae-Kyung Kwak, Dae-Hwan Kim, Hyung-Min Lee, Ji-Hyo Hwang, Je-Hyun Yoo

**Affiliations:** 1 Department of Orthopaedic Surgery, Hallym University Sacred Heart Hospital, Hallym University College of Medicine, 896 Pyeongchon-dong, Dongan-gu, Anyang, 431-070 Republic of Korea; 2 Department of Orthopaedic Surgery, Kangnam Sacred Heart Hospital, Hallym University College of Medicine, Seoul, Republic of Korea

**Keywords:** Proximal femur fracture, Cephalomedullary nailing, Antiplatelet, Surgical outcome

## Abstract

**Background:**

Antiplatelet medication has been frequently performed in elderly patients with hip fracture, because of comorbidities. This observational cohort study was to evaluate the effect of continuous perioperative antiplatelet medication on the outcomes after cephalomedullary nailing (CMN) in elderly patients with a proximal femur fracture.

**Methods:**

One hundred and sixty-two consecutive patients aged ≥70 years undergoing CMN for proximal femur fracture between January 2015 and January 2017 were recruited. Of the 162 patients, 47 (study group) taking antiplatelets preoperatively due to comorbidities were compared with 107 (control group) who were not on antiplatelets. 8 patients taking anticoagulant medication were excluded. Postoperative hemoglobin (Hb) and hematocrit (Hct) levels, transfusion amount and estimated blood loss (EBL), occurrence of venous thromboembolism (VTE) and delirium, intensive care unit (ICU) admission, complications, length of hospital stay, readmission, and in-hospital and 1-year mortalities were measured and compared between the two groups.

**Results:**

A higher number of patients in the study group had concomitant cardiovascular (*p* = 0.006) and endocrinologic (*p* = 0.004) diseases, received perioperative transfusion (*p* = 0.003), and were admitted to ICU postoperatively (*p* = 0.014). However, there were no significant differences in postoperative Hb and Hct levels, EBL, length of hospital stay, and the incidences of VTE and delirium between the two groups. In addition, in-hospital and 1-year mortalities as well as postoperative complications showed no significant differences between both groups.

**Conclusions:**

CMN can be performed without delay in elderly patients with proximal femoral fracture receiving antiplatelet therapy prior to admission without discontinuing antiplatelets, and is as safe as in patients who are not on antiplatelet medication. However, more caution is required with respect to transfusions and ICU care after surgery in these patients.

**Electronic supplementary material:**

The online version of this article (10.1186/s12891-019-2504-5) contains supplementary material, which is available to authorized users.

## Background

Osteoporotic hip fractures have become a substantial cause of morbidity and mortality in the aging population [1]. As the average lifespan has increased, the incidence of hip fractures in this aging population is expected to increase further. In fact, the incidence of osteoporotic hip fractures is steadily increasing, and is predicted to reach 4.5 million individuals worldwide per year by 2050 [2].

Over 65% of hip fracture patients have an American Society of Anesthesiologists (ASA) physical status classification of 3 or above, which reflects the high prevalence of coexisting comorbidities [3]. Therefore, a significant proportion of elderly patients are on antiplatelet therapy, when presenting with a hip fracture. Aspirin is a well-known antiplatelet agent used for the prevention of cardiovascular and cerebrovascular events. Clopidogrel is another potent antiplatelet drug that inhibits adenosine dinucleotide phosphate receptors, and its potency for secondary prevention after cardiovascular events is considered superior to aspirin [4]. Dual antiplatelet therapy (aspirin and clopidogrel) is especially effective in preventing vascular events and associated mortality; however, this regimen may significantly increase the risk of bleeding [5].

In elderly patients with hip fracture receiving antiplatelet therapy prior to admission, it may be difficult for surgeons to decide on the timing of surgery and whether to continue or discontinue perioperative antiplatelet medication. To minimize the blood loss perioperatively, surgery for hip fracture in patients receiving antiplatelet therapy is often delayed to perform an antiplatelet washout. The potential risk of hematoma formation associated with regional anesthesia in orthopaedic patients reinforces the rationale for interruption in antiplatelet therapy, leading to a subsequent delay in surgery [6]. Meanwhile, acute discontinuing of antiplatelet agents in patients with hip fracture before the surgery may cause a rebound effect and induce thromboembolic events in patients with atherosclerosis [5, 7]. Surgical delay in hip fracture management of more than 2 days is associated with a significantly increased risk of complications and mortality within 30 days and at 1 year [8, 9].

Early surgical intervention after admission in elderly patients with hip fracture is usually recommended if there are no safety issues. However, there is a wide variation in the management of elderly patients with hip fracture who are on long-term antiplatelet therapy in terms of the discontinuation of antiplatelets and the timing of surgery, with a lack of consensus on these issues amongst surgeons.

This cohort study was conducted to investigate the effect of continuing perioperative antiplatelet medication on surgical outcomes after cephalomedullary nailing (CMN) without a surgical delay in elderly patients with proximal femoral fracture.

## Methods

Ethics approval was obtained from our institutional review board. A prospectively compiled database was used to recruit patients aged ≥70 years who underwent CMN for proximal femoral fracture at our institute between January 2015 and January 2017. We excluded patients with polytrauma, pathological fractures, coagulopathies, hematological malignancies, thrombocytopenia on admission (platelet count < 150 × 10^9^/L), recent active bleeding, and gastrointestinal ulcers. During this period, 162 patients were identified. Most patients underwent index surgery within 2 days and no longer than 3 days after admission to our hospital. The exceptions were specific patients with severe systemic comorbidities threatening life, for whom a longer time was taken to optimize the patients’ medical conditions by further evaluation and appropriate preoperative management.

**Fig. 1:**
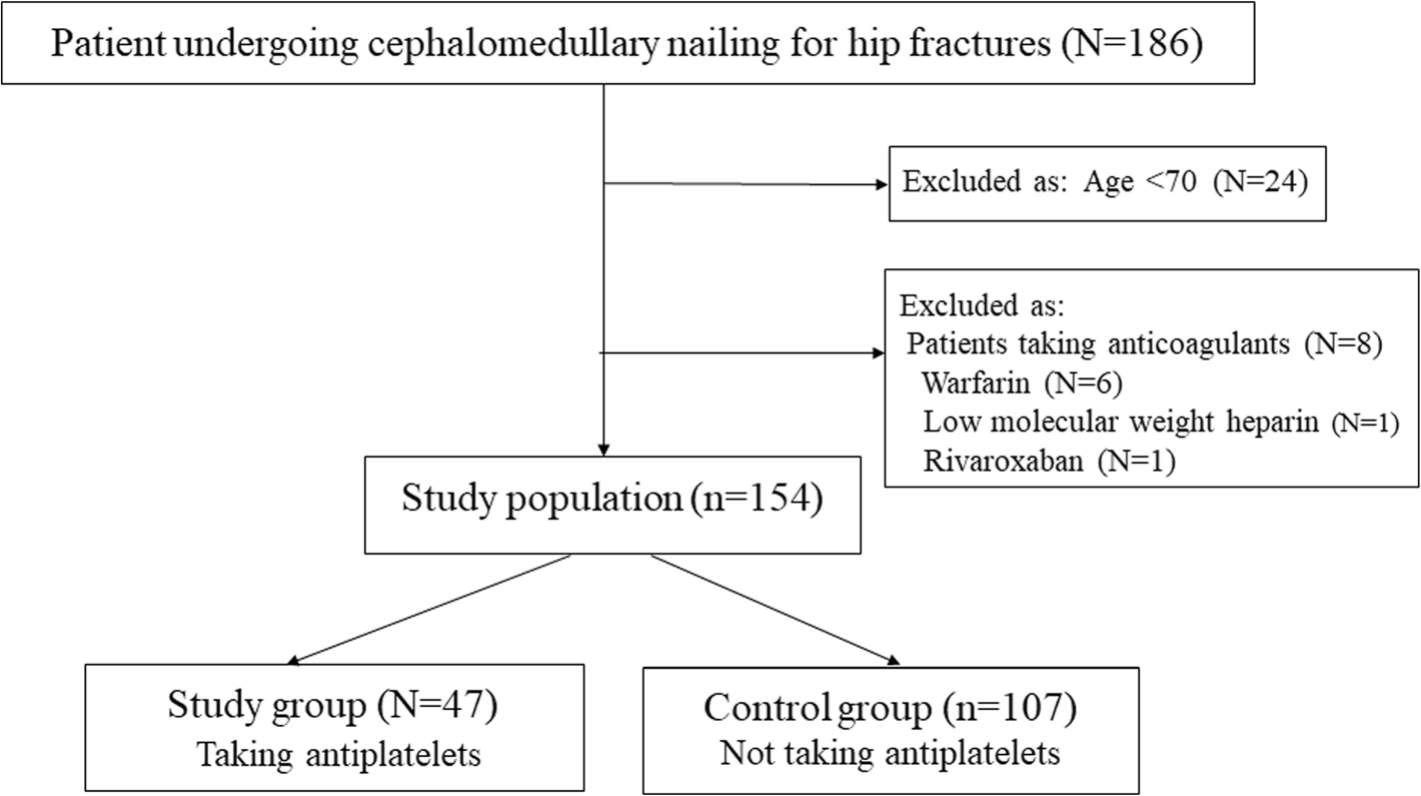
Flow chart showing the enrollment of patients for this study

Of the total of 162 patients, 8 receiving anticoagulant therapy such as warfarin and heparin were excluded, and 154 were finally enrolled in this study. The enrolled patients were divided into two groups (Fig. 1). Forty-seven patients in the study group had received antiplatelet therapy prior to being injured due to comorbidities such as cardiovascular or cerebrovascular diseases and the remaining (107 patients) in the control group had not received antiplatelet therapy. The study group consisted of 29 patients on clopidogrel, 13 on aspirin, 4 on aspirin and clopidogrel, and 1 on a platelet aggregation inhibitor (Triflusal).

Each patient underwent imaging studies including x-ray and 3-dimensional CT for detailed assessment of fracture pattern before the surgery. Multidisciplinary consultations were also conducted for all patients to assess the operation risk and to optimize medical conditions. At admission, two types of mechanical thromboprophylaxis, intermittent pneumatic compression device (IPCD) and graduated compression stockings (GCSs), were implemented in all patients. There was no additional chemical prophylaxis preoperatively. The study group of patients taking antiplatelet agents preinjury continued to take antiplatelet medication without discontinuation from the day of admission until the day of surgery.

All operations were performed by one experienced orthopaedic surgeon in our institute. Surgical wound drainage was not used in any patient. All patients in both groups received mechanical thromboprophylaxis using IPCD and GCSs until discharge (for about two weeks). In the control group, a once-daily subcutaneous administration of 40 mg enoxaparin was commenced 24 h after the index surgery and was continued for 10 to 14 days as combined chemical prophylaxis, according to our protocol. The study group took antiplatelet agents continuously before and after the surgery.

Postoperative rehabilitation that focused on early mobilization was implemented according to our protocol. Standing with weight-bearing as tolerated and ambulation using a walker were initiated at around 2 to 3 days postoperatively. Most patients were admitted for approximately 12 days after surgery because the National Public Health System and private health insurance companies cover most of the costs in our country. These patients were then transferred to the Department of Rehabilitation in our institute, affiliated rehabilitation centers, or nursing facilities for continuous rehabilitation lasting approximately two to three weeks. Postoperatively, the patients were followed up at 3, 6, and 12 months for a minimum of 12 months, and yearly thereafter. Patients who had difficulty attending the outpatient clinic were followed up through telephone consultation. Details regarding the date and cause of death were verified by medical record reviews and direct or telephone interviews of the patients’ families by independent researchers.

Demographic data such as gender, age, body mass index (BMI), bone mineral density (BMD), ASA score, the time from admission to operation, preoperative hemoglobin (Hb) and Hematocrit (Hct), and comorbidities were collected from the electronic patient records of our hospital. Data on comorbid medical conditions were categorized based on the presence of the following: cardiovascular, pulmonary, endocrinologic, neurologic, nephrologic, and hemato-oncologic diseases. In addition, perioperative data were collected, including postoperative changes of Hb and Hct levels, total amount of transfusion, blood volume (BV), estimated blood loss (EBL), the type of anesthesia, and operation time. EBL was calculated using Mercuriali’s formula [10], which is based on the Hct preoperatively and Hct on the fifth postoperative day. This formula requires the patient’s BV, so Nadler’s formula [11] was additionally used for BV estimation [see Additional file 1].

Postoperative data including the occurrence of deep vein thrombosis (DVT), pulmonary embolism (PE), and delirium, intensive care unit (ICU) admission and ICU period, length of hospital stay, re-admission, in-hospital and 1-year mortality rates, and occurrence of medical and surgical complications were also analyzed.

### Statistical analysis

The statistical package SPSS version 17 (SPSS Inc., Chicago, IL) was used for statistical analysis. Demographic data as well as peri- and postoperative data were compared by using the Student t-test for continuous variables and the chi-square test for dichotomous variables. Fisher’s exact test was used when expected counts were less than 5. Statistical significance was determined by obtaining a *p* < 0.05 in all the analyses.

## Results

The demographic and preoperative data of patients in the two groups are presented in Table 1. The demographic data including age, gender, BMI, BMD, ASA grade, time to operation, and preoperative Hb and Hct showed no differences in both groups. However, for patients with other medical comorbidities, the study group had more significant rate of cardiovascular disease (95.7% vs 77.6%, *p* = 0.006) and endocrinologic disease (51.1% vs 27.1%, *p* = 0.004) compared to the control.

**Table 1:** Demographic and clinical characteristics between two groups

Variables	Control group (*n* = 107)	Study group (*n* = 47)	*p*-value
Age (years)	81.5 ± 7.0	81.5 ± 6.3	0.971
Gender (male: female)	32: 75	11: 36	0.408
Time to operation (days)	2.7 ± 3.6	2.3 ± 1.5	0.546
ASA grade			0.405
II	4	0	
III	94	43	
IV	9	4	
BMI (kg/m^2^)	21.7 ± 4.0	22.2 ± 4.1	0.524
BMD (T-score)	−3.04 ± 1.15	−2.91 ± 1.03	0.535
Preoperative Hb (g/dL)	11.2 ± 1.5	10.8 ± 1.3	0.201
Preoperative Hct (%)	33.3 ± 4.5	32.6 ± 3.5	0.323
Underlying disease			
Cardiovascular	83(77.6%)	45(95.7%)	**0.006**
Endocrinologic	29(27.1%)	24(51.1%)	**0.004**
Pulmonary	10(9.3%)	3(6.4%)	0.755
Neurologic	27(25.2%)	12(25.5%)	0.969
Nephrologic	11(10.3%)	6(12.8%)	0.650
Hemato-oncologic	15(14.0%)	6(12.8%)	0.835

Continuous variables are presented as mean ± standard deviation

*ASA* American Society of Anesthesiologists, *BMI* Body mass index, *BMD* bone mineral density, *Hb* hemoglobin, *Hct* hematocrit

Bold indicates a statistically significant value (< 0.05)

During the perioperative period, there were no significant differences in operation time, the type of anesthesia, BV, EBL, and the postoperative changes of Hb and Hct levels in both groups. However, the total transfusion volume was significantly greater in the study group on antiplatelet medication (*p* = 0.003) (Table 2).

**Table 2:** Perioperative data between two groups

Variables	Control group (*n* = 107)	Study group (*n* = 47)	*p*-value
POD 1 Hb	9.8 ± 1.1	9.7 ± 1.2	0.362
POD 3 Hb	9.4 ± 0.9	9.3 ± 0.9	0.506
POD 5 Hb	9.7 ± 1.0	9.7 ± 1.0	0.748
POD 5 Hct	29.2 ± 3.2	29.3 ± 2.9	0.903
Total transfusion (ml)	695.3 ± 487.5	956.6 ± 519.5	**0.003**
Blood volume* (ml)	3466.9 ± 641.9	3272.5 ± 551.8	0.073
Estimated blood loss* (ml)	393.0 ± 211.8	443.4 ± 179.3	0.158
Anesthesia (general: spinal)	79: 28	39: 8	0.217
Operation time (min)	61.8 ± 27.7	66.7 ± 39.8	0.383

Continuous variables are presented as mean ± standard deviation

*POD* postoperative day, *Hb* hemoglobin, *Hct* hematocrit

*Appendix 1

Bold indicates a statistically significant value (< 0.05)

With respect to postoperative data and complications, there were no significant differences in the occurrence of DVT, PE, and delirium, length of hospital stay, readmission, and in-hospital and 1-year mortality rates between the two groups. In addition, the occurrence of medical and surgical complications showed no differences between both groups (Table 3). However, more patients in the study group than in the control group were admitted to ICU after surgery due to their comorbidities (*p* = 0.014). Nevertheless, there was no difference in the duration of ICU stay between both groups.

**Table 3:** Postoperative complications between two groups

Variables	Control group (*n* = 107)	Study group (*n* = 47)	*p*-value
Deep vein thrombosis	1 (0.9%)	1 (2.1%)	0.519
Pulmonary embolism	2 (1.9%)	0 (0%)	0.345
Delirium	49 (45.8%)	23 (48.9%)	0.729
ICU admission	43 (40.2%)	29 (59.2%)	**0.014**
ICU period (days)	2.6 ± 2.0	2.7 ± 2.0	0.894
Length of hospital stay (days)	22.9 ± 9.3	25.9 ± 14.7	0.123
Readmission	33 (30.8%)	16 (34.0%)	0.694
In-hospital mortality	4 (3.7%)	1 (2.1%)	1.000
1-year mortality	15 (14.0%)	7 (14.9%)	0.886
Complications			
Medical	68 (63.5%)	29 (59.2%)	0.602
Cardiovascular	4	1	
Pulmonary	24	10	
Neurologic	1	1	
Nephrologic	16	10	
Hematologic	6	2	
Urologic	17	5	
Surgical	0 (0%)	0 (0%)	

Continuous variables are presented as mean ± standard deviation

Bold indicates a statistically significant value (< 0.05)

## Discussion

Elderly patients with hip fracture frequently receive antiplatelet therapy due to their multiple comorbidities, including cardiovascular disease [4]. The current study demonstrated that the continuation of antiplatelet medication during the perioperative period in these patients result in similar outcomes after CMN for proximal femoral fractures as in patients who were not on antiplatelet therapy. There were no significant differences between the two groups with respect to perioperative results, postoperative medical and surgical complications, readmission, and in-hospital and 1-year mortality. Only total transfusion volume and ICU admission showed a significant increase in elderly patients on continuous perioperative antiplatelet therapy. However, the lengths of ICU and hospital stay after the admission showed no significant differences between the two groups. We believe that the higher rate of ICU admission is related to comorbidities in patients of the study group on continuous perioperative antiplatelet medication, which make them on that medication and require close monitoring postoperatively compared to the control group not on antiplatelet medication.

The incidence of hip fractures have increased as the population has aged [12]. Due to the high incidence of cardiovascular or cerebrovascular comorbidities in geriatric population, many patients have received long-term antiplatelet treatment [3]. Aspirin ingestion was found to increase perioperative blood transfusion requirements in hip fracture surgery [13]. Grujic and Martin [14] showed that clopidogrel treatment was associated with a 37-fold risk for reoperation after general, orthopaedic, vascular or cardiothoracic surgery, and that the effect was reduced but still present in patients in whom treatment was stopped for 7 days prior to surgery. Furthermore, the platelet aggregation is fully reactivated 10 days after withdrawing from antiplatelet therapy [5]. However, withdrawal of antiplatelet agents in patients with hip fracture prior to operation may induce a rebound effect and potentially lead to thromboembolic events in patients with atherosclerosis [5, 7]. Discontinuation of clopidogrel and delay in surgical intervention for hip fractures are also linked to high rates of DVT and PE [15]. Furthermore, delaying surgical intervention for proximal femoral fractures has been reported to increase perioperative complications and mortality [9]. Therefore, surgeons may find it difficult to make decisions about the timing of surgery and whether antiplatelet medication should be continued or discontinued in individual elderly patients. Moreover, there is a lack of uniformed consensus among surgeons regarding these issues.

At our institution, elderly patients with hip fracture underwent surgical management as soon as they were medically optimized according to the same protocol for patients who are not on antiplatelet therapy, with the rationale to reduce perioperative complications and mortality associated with surgical delay. However, the surgeon must prudently consider the risks and benefits of this policy in each patient. For elderly patients with proximal femoral fracture treated with CMN according to this policy, we conducted the present study to compare patients on continuous antiplatelet therapy and those who were not on antiplatelets, and to evaluate the effect of continuing antiplatelet therapy during the perioperative period on outcomes after the surgery.

Chechik et al. [16] in their matched cohort study reported that patients receiving antiplatelet drugs can safely undergo hip fracture surgery without delay, regardless of greater perioperative blood loss and thrombo-embolic or postoperative bleeding events. However, their study involved patients aged > 40 years including elderly patients, and comprised patients with all intracapsular and extracapsular hip fractures individually treated with three procedures based on the fracture pattern; proximal femoral nailing, dynamic hip screw plating, and hemiarthroplasty. We believe that their study design with the selection of patient age group and fracture pattern makes their conclusion less representative. The results between elderly patients with comorbidities and relatively healthy young patients may vary and several fracture types and procedures may cause bias to the results. Collinge et al. [17] reported that 74 patients taking clopidogrel who underwent early hip fracture surgery were not at a substantially higher risk for bleeding and bleeding complications, and did not have a higher mortality rate, than 619 patients not on clopidogrel. However, clopidogrel medication was stopped at the time of admission in most of the patients and restarted the therapy after surgery. Furthermore, the results might have been affected by the fact that they did not report the type of anesthesia. Doleman and Moppett [18] reported in a meta-analysis that early hip fracture surgery appears safe with similar mortality rates between patients on clopidogrel and those not on clopidogrel, although there may be a small increase in the rate of blood transfusion. Although our findings agree with their study, there is no consistent guideline across studies thus far on whether to discontinue antiplatelet drugs at admission and restart postoperatively, or continue antiplatelets perioperatively in elderly patients with hip fracture. In addition, most of these studies enrolled relatively young patients and the elderly, as well as patients treated with various surgical procedures according to the fracture type. Meanwhile, our study was aimed solely at elderly patients (≥ 70 years) treated only with minimally invasive method of CMN for extracapsular proximal femoral fractures for the two groups matched for age, gender, ASA grade, the time to operation, anesthesia type, and operation time. Under these conditions, the current study demonstrated that early surgery with continuing perioperative antiplatelet medication is safe and has no negative effect on surgical outcomes if more attention is paid to perioperative transfusion and ICU care for these patients. We believe that this design of the current study strengthens our results when compared with previous studies.

Zhang et al. [19] reported that continuation of preoperative clopidogrel for patients undergoing intramedullary nailing for an intertrochanteric fracture resulted in higher chances of intraoperative transfusion, increased ICU admission and total duration of hospitalization, and a lower one-year survival rate, leading to poor prognosis. Our results were consistent with those of Zhang et al. in terms of total transfusion volume and ICU admission rates. However, in our study, there were no significant differences in the lengths of ICU and hospital stay, the occurrence of postoperative complications, and in-hospital and 1-year mortality rates between the two groups. This difference could be due to the inclusion of patients on other antiplatelet drugs such as aspirin, which are less potent than clopidogrel. However, our study was aimed at patients older than those enrolled in the study by Zhang et al. with better matching of two groups. Subsequently, similar results were observed between these two groups treated with CMN according to the same protocol.

Our study is limited by its retrospective design and relatively small sample size. We did, however, identify consecutive elderly patients over 70 years of age undergoing only CMN according to the same protocol from admission to discharge regardless of antiplatelet medication prior to admission, and formed two well-matched groups. Second, as this study was aimed at geriatric hip fracture patients on only antiplatelet agents, additional research should be conducted using other anticoagulants that have a different mechanism of action from antiplatelets.

Nevertheless, our study has its strengths as it enrolled consecutive hip fracture patients over 70 years of age, undergoing only one procedure (CMN) using the same implant, managed according to the same protocol by one experienced surgeon in a single center. Therefore, the potential for bias due to variations in several diagnoses and procedures performed is expected to be minimal, and our results would have allowed us to draw significant conclusions. Second, both groups in our study were well matched with respect to gender, age, ASA grade, time to operation after admission, BMI, preoperative Hb and Hct levels, anesthesia, and operation time. The mean age was over 80 years with high prevalence of ASA score 3 in both groups. Third, the scope of this study included addressing issues related specifically to the continuation of antiplatelet drugs and early surgery regardless of antiplatelet medication in elderly patients. Finally, this study compared perioperative outcomes including estimated blood loss and transfusion, postoperative complications, and readmission and mortality rates between the two matched groups.

The results of this study contribute to the body of evidence that hip fracture surgery can be performed in elderly patients without interruption of ongoing antiplatelet therapy prior to admission as safely and promptly as in patients not receiving antiplatelet therapy. In the future, well-controlled prospective studies or large multicenter comparative studies involving a large number of patients are recommended to corroborate the results of this study.

## Conclusions

In elderly patients with proximal femoral fracture receiving antiplatelet therapy prior to injury, continuing antiplatelet agents perioperatively was observed to have higher total transfusion volume and ICU admission rate than in patients not on antiplatelet medication. However, other complications and mortality rate were not significantly affected. Therefore, in elderly patients with proximal femoral fracture receiving antiplatelets due to comorbidities, prompt surgery can be safely performed with no interruption of perioperative antiplatelet therapy, although more attention should be paid with respect to the need for perioperative transfusion and ICU care after surgery.

## Additional file

Additional file 1: Formulas used for calculation of EBL. (DOCX 14 kb)

## Abbreviations

ASA: American Society of Anesthesiologists
BMD: Bone mineral density
BMI: Body mass index
BV: Blood volume
CMN: Cephalomedullary nailing
DVT: Deep vein thrombosis
EBL: Estimated blood loss
GCSs: Graduated compression stockings
Hb: Hemoglobin
Hct: Hematocrit
ICU: Intensive care unit
IPCD: Intermittent pneumatic compression device
PE: Pulmonary embolism
VTE: Venous thromboembolism
