# Supplementary material for: Perioperative antiplatelet in elderly patients aged over 70 years treated with proximal femur fracture: continue or discontinue?
Source: BMC Musculoskelet Disord. 2019 Mar 25;20:124. doi: 10.1186/s12891-019-2504-5 (PMC6434804; doi:10.1186/s12891-019-2504-5)
Supplement: Supplementary file 1 — Formulas used for calculation of EBL. (DOCX 14 kb) [file 12891_2019_2504_MOESM1_ESM.docx]

**Formulas used for calculation of EBL**

(A) Blood volume (ml) = 604 + 0.0003668 × [Height (cm)]^3^ + 32.2 × weight (kg) for male

(B) Blood volume (ml) = 183 + 0.000356 × [Height (cm)]^3^ + 33 × weight (kg) for female

(C) EBL (ml) = blood volume (ml) × (Hct _preoperative_ –Hct _postoperative day 5_ + ml of transfused RBC

(A) Nadler’s formula for male’s blood volume estimation; (B) Nadler’s formula for female’s blood volume estimation; (C) Mercuriali formula for estimated blood loss calculation. The hematocrits must be written as decimal fractions.
